# Altered Ureteric Branching Morphogenesis and Nephron Endowment in Offspring of Diabetic and Insulin-Treated Pregnancy

**DOI:** 10.1371/journal.pone.0058243

**Published:** 2013-03-13

**Authors:** Stacey N. Hokke, James A. Armitage, Victor G. Puelles, Kieran M. Short, Lynelle Jones, Ian M. Smyth, John F. Bertram, Luise A. Cullen-McEwen

**Affiliations:** 1 Department of Anatomy and Developmental Biology, Monash University, Clayton, Victoria, Australia; 2 Department of Biochemistry and Molecular Biology, Monash University, Clayton, Victoria, Australia; 3 School of Medicine (Optometry), Deakin University, Waurn Ponds, Victoria, Australia; The University of Manchester, United Kingdom

## Abstract

There is strong evidence from human and animal models that exposure to maternal hyperglycemia during *in utero* development can detrimentally affect fetal kidney development. Notwithstanding this knowledge, the precise effects of diabetic pregnancy on the key processes of kidney development are unclear due to a paucity of studies and limitations in previously used methodologies. The purpose of the present study was to elucidate the effects of hyperglycemia on ureteric branching morphogenesis and nephrogenesis using unbiased techniques. Diabetes was induced in pregnant C57Bl/6J mice using multiple doses of streptozotocin (STZ) on embryonic days (E) 6.5-8.5. Branching morphogenesis was quantified *ex vivo* using Optical Projection Tomography, and nephrons were counted using unbiased stereology. Maternal hyperglycemia was recognised from E12.5. At E14.5, offspring of diabetic mice demonstrated fetal growth restriction and a marked deficit in ureteric tip number (control 283.7±23.3 vs. STZ 153.2±24.6, mean±SEM, *p*<0.01) and ureteric tree length (control 33.1±2.6 mm vs. STZ 17.6±2.7 mm, *p* = 0.001) vs. controls. At E18.5, fetal growth restriction was still present in offspring of STZ dams and a deficit in nephron endowment was observed (control 1246.2±64.9 vs. STZ 822.4±74.0, *p<*0.001). Kidney malformations in the form of duplex ureter and hydroureter were a common observation (26%) in embryos of diabetic pregnancy compared with controls (0%). Maternal insulin treatment from E13.5 normalised maternal glycaemia but did not normalise fetal weight nor prevent the nephron deficit. The detrimental effect of hyperglycemia on ureteric branching morphogenesis and, in turn, nephron endowment in the growth-restricted fetus highlights the importance of glycemic control in early gestation and during the initial stages of renal development.

## Introduction

The global prevalence of diabetes in pregnancy is increasing, both in terms of gestational diabetes mellitus (first diagnosis of diabetes in pregnancy) and pre-existing type 1 and 2 diabetes mellitus [1], [2]. Irrespective of the etiology of maternal diabetes, the developing fetus is exposed to a hyperglycemic intrauterine environment, which confers an increased risk of adverse perinatal outcomes such as birth trauma, cesarean delivery and altered fetal growth patterns [3], [4]. Maternal insulin therapy can reduce the likelihood of these adverse perinatal outcomes [5]. Offspring exposed to intrauterine hyperglycemia also have an elevated risk of developing congenital malformations [6], [7], [8], and moreover, have an increased susceptibility to develop metabolic diseases in adulthood [9], [10], [11].

Maternal hyperglycemia is also associated with altered offspring kidney development. Although kidney development is a complicated process, two key events in kidney development are ureteric branching morphogenesis and nephrogenesis [12]. The kidney arises by the outgrowth of the ureteric bud and branching of the ureteric epithelium into a complex tree-like structure, which ultimately forms the renal collecting duct system and calyces. Nephrogenesis is induced only at the tips of the branching ureteric epithelium highlighting the association between ureteric tip number and nephron endowment. Of the congenital malformations observed in offspring of diabetic pregnancy, genitourinary defects and renal malformations are prevalent [13], [14], [15]. Studies of streptozotocin (STZ) induced type 1 diabetes mellitus (T1DM) suggest that exposure to hyperglycemia can lead to reduced nephron endowment [16], [17], [18]. However, studies are few and less than optimal methods have been used to count glomeruli. While nephron endowment is highly dependent on adequate ureteric branching morphogenesis, to date the effect of hyperglycemia on ureteric tree development has not been assessed *in vivo*. Until recently, assessment of ureteric branching morphogenesis typically involved culturing fetal kidneys for a number of days *in vitro*. This procedure results in a flattened kidney that can be wholemount immunostained and imaged. Previous *in vitro* culture studies have been limited to measures of ureteric tip number and have produced inconsistent results [16], [19], [20]. These discrepancies are likely the result of variability inherent to culture preparations, differences in the length of culture time and the type of media and supplements used. A better understanding of the effect of hyperglycemia on ureteric branching morphogenesis, and in turn nephrogenesis, is required.

To explore the effect of hyperglycemia on the developing kidney we utilized unbiased techniques to determine the effect of maternal diabetes on key processes of kidney development in the mouse. Ureteric branching morphogenesis was quantified *ex vivo* using Optical Projection Tomography (OPT) and nephron number was estimated using the gold standard stereological method [21]. Insulin was administered to pregnant dams to determine if glycemic control during a period of early nephrogenesis could prevent a nephron deficit in offspring of diabetic pregnancy.

## Results

### STZ increases maternal blood glucose concentrations from E12.5 leading to offspring growth restriction and reduced ureteric tree development at E14.5

**Figure 1:**
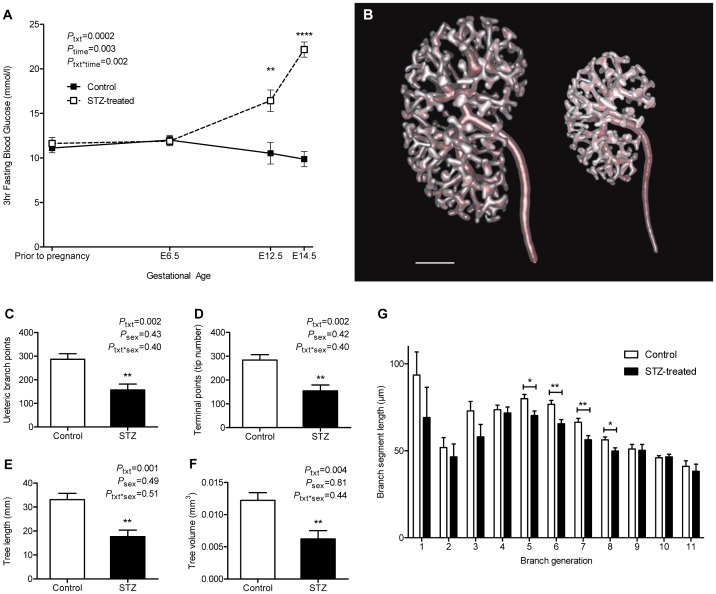
Maternal blood glucose concentrations and ureteric tree development in offspring of the E14.5 cohort. (A) 3 hour fasting blood glucose concentration of control (solid line) and STZ-treated (dashed line) dams throughout gestation. (B) Rendered reconstructions of the ureteric tree of control (left) and STZ-treated (right) embryos at E14.5. Scale bar denotes 200µm. Quantitative analysis of (C) ureteric branch points, (D) branch tip number, (E) ureteric tree length, (F) ureteric tree volume and (G) branch segment length of control (clear bar) and STZ-treated (solid bar) embryos. Glucose analysis by repeated measures two-way ANOVA for maternal STZ treatment and time followed by Fishers LSD post hoc analysis; n = 5 dams/group. Ureteric tree analysis by two-way ANOVA for maternal STZ treatment and offspring sex followed by Fishers LSD post-hoc analysis; n =  5 litters comprising 9 kidneys per group. Values are mean±SEM. *p<0.05, **p<0.01, ****p<0.0001 *vs*. control group.

Maternal blood glucose concentrations did not differ between control dams (n = 5) and STZ-treated dams (n = 5) before pregnancy (*p* = 0.50) or prior to STZ injections at E6.5 (*p* = 0.88) (**Figure 1A**). Maternal blood glucose concentrations were significantly elevated post STZ injections at E12.5 (*p*<0.01) and E14.5 (*p*<0.0001). Glucose area under the curve (AUC) was greater in STZ-treated dams than in control dams across gestation (control 168.82±8.96 mmol/l.day *vs*. STZ 205.76±4.99 mmol/l.day *p*<0.01).

Amniotic fluid of E14.5 STZ-treated embryos had a significantly greater glucose concentration compared with that of control embryos (control 7.16±0.49 mmol/l *vs*. STZ 16.30±1.49 mmol/l, *p<*0.0001).

There was no difference in litter size (control 8.2±0.7 embryos *vs*. STZ 8.6±0.4 embryos, *p* = 0.62), viable embryos (control 8.2±0.7 embryos *vs*. STZ 6.6±0.5 embryos, *p* = 0.09) or resorptions (control 0.4±0.2 embryos *vs*. STZ 0.8±0.4 embryos, *p* = 0.40) between control and diabetic pregnancies at E14.5. Dead embryos were absent in control pregnancies however 23% of embryos of diabetic pregnancies were dead at collection.

Embryos of STZ-treated dams were 32% lighter in weight (*p*<0.01), 12% smaller in crown-rump length (*p*<0.01) and 10% smaller in head width (*p* = 0.01) compared with control embryos at E14.5 (**Table 1**). Embryos of diabetic dams were not developmentally delayed, as assessed by Theiler staging. Placentas of STZ-treated embryos were significantly lighter than placentas of control embryos at E14.5 (75.43±5.18 mg; 115.19±5.09 mg, *p* = 0.001). While no treatment*sex interaction was found across growth parameters, sex had a significant effect on growth with male offspring exhibiting heavier bodyweights (*p*<0.01) and placental weights (*p* = 0.02) than females.

**Table 1:** Growth parameters of control and STZ-treated embryos at E14.5; and control, STZ and STZ+Insulin-treated embryos at E18.5.

			Bodyweight (mg)	Crown-Rump Length (mm)	Head width (mm)	Placenta weight (mg)
**E14.5**	**Control**	**Male**	280.3±15.7	11.9 ±0.3	7.1±0.2	118.8±5.5
		**Female**	261.1±15.8	11.8±0.3	7.0±0.2	111.6±5.6
	**STZ**	**Male**	189.9±15.9	10.5±0.3	6.4±0.2	79.9±5.7
		**Female**	180.1 ±16.1	10.4±0.3	6.3±0.2	71.0±5.8
	***P*** **_treatment_**		**0.004**	**0.004**	**0.01**	**0.001**
	***P*** **_sex_**		**0.008**	**0.32**	**0.58**	**0.02**
	***P*** _treatment*sex_		**0.38**	**0.87**	**0.63**	**0.79**
**E18.5**	**Control**	**Male**	1169.7±32.0	20.9±0.5	10.8±0.2	112.6±5.5
		**Female**	1184.4±33.7	21.0±0.5	10.9±0.2	108.7±5.6
	**STZ**	**Male**	748.7±37.8	16.8±0.5	9.3±0.2	72.9±6.3
		**Female**	727.7±40.3	16.6±0.6	9.4±0.2	61.4±7.0
	**STZ+Insulin**	**Male**	752.3±48.9	17.0±0.7	10.0±0.3	80.3±8.3
		**Female**	713.7±55.4	16.2±0.8	9.6±0.3	69.7±9.0
	***P*** **_treatment_**		**<0.0001**	**<0.0001**	**<0.0001**	**<0.0001**
	***P*** **_sex_**		**0.54**	**0.12**	**0.50**	**0.04**
	***P*** _treatment*sex_		**0.61**	**0.19**	**0.27**	**0.62**

Data from E14.5 and E18.5 cohorts were analysed separately by a linear mixed model with maternal STZ and insulin treatment and offspring sex as independent variables, weighted for litter. Values are mean±SEM. E14.5: (n)  =  control (n =  5 litters comprising 41 embryos), STZ-treated (n = 5 litters comprising 33 embryos). E18.5: (n)  =  control (n = 11 litters comprising 72 embryos), STZ (n = 10 litters comprising 41 embryos), STZ+Insulin (n = 5 litters comprising 25 embryos).

OPT demonstrated that the kidneys of E14.5 embryos exposed to hyperglycemia were smaller than control kidneys and had a significantly smaller ureteric tree (**Figure 1B**). Quantitative analysis revealed that the kidneys of embryos exposed to hyperglycemia contained 45% fewer ureteric branch points (*p* = 0.002; **Figure 1C**), 46% fewer ureteric tips (*p* = 0.002; **Figure 1D**), a 47% reduction in total ureteric tree length (*p* = 0.001; **Figure 1E**) and a 49% reduction in tree volume (*p* = 0.004; **Figure 1F**) compared with control kidneys. Kidneys of STZ-treated embryos had on average fewer branch generations than control kidneys (control 7.5±0.1 vs. STZ 6.7±0.2, *p* = 0.001). Compared with controls, STZ-treated kidneys had significantly shorter branch segment lengths from branch generations five to eight (5^th^ generation, *p* = 0.02; 6^th^ generation, *p* = 0.004; 7^th^ generation, *p* = 0.008; 8^th^ generation, *p* = 0.02; **Figure 1G**).

Spearman's rank coefficient revealed bodyweight, STZ treatment and maternal glucose AUC to have strong associations with measures of ureteric tree development at E14.5 (**Table 2**). In a stepwise multiple regression model, fetal bodyweight, maternal STZ treatment and maternal glucose AUC predicted branch point number (R^2^ = 0.87, *p*<0.0001), with bodyweight contributing the most to the model (β = 1.03, *p*<0.0001) above maternal STZ treatment (β = 0.29, *p* = 0.17) and maternal glucose AUC (β = −0.18, *p* = 0.29). When variables were considered independently, fetal body weight was the only statistically significant factor (R^2^ = 0.85, *p*<0.0001). Similar trends were observed for tip number, tree length, tree volume and average branch generations.

**Table 2:** Spearman's rank coefficients: associations between measures of ureteric tree development and independent variables at E14.5.

	Bodyweight	Treatment	Glucose AUC
**Treatment**	−0.87		
	(*p*<0.01)		
**Glucose AUC**	−0.82	0.87	
	(*p*<0.01)	(*p*<0.01)	
**Branch number**	0.87	−0.82	−0.81
	(*p*<0.01)	(*p*<0.01)	(*p*<0.01)
**Tip number**	0.87	−0.82	−0.81
	(*p*<0.01)	(*p*<0.01)	(*p*<0.01)
**Tree length**	0.87	−0.82	−0.78
	(*p*<0.01)	(*p*<0.01)	(*p = *0.01)
**Tree volume**	0.87	−0.82	−0.78
	(*p*<0.01)	(*p*<0.01)	(*p = *0.01)
**Average number of branch generations**	0.84	−0.76	−0.72
	(*p*<0.01)	(*p<*0.01)	(*p<*0.01)

Spearman's Rho (top value), *p* value (bottom value). Negative values indicate a negative association. AUC  =  area under the curve.

### Administration of insulin from E13.5 reduces maternal blood glucose concentrations by E15.5 but does not normalise fetal growth or nephron number

**Figure 2:**
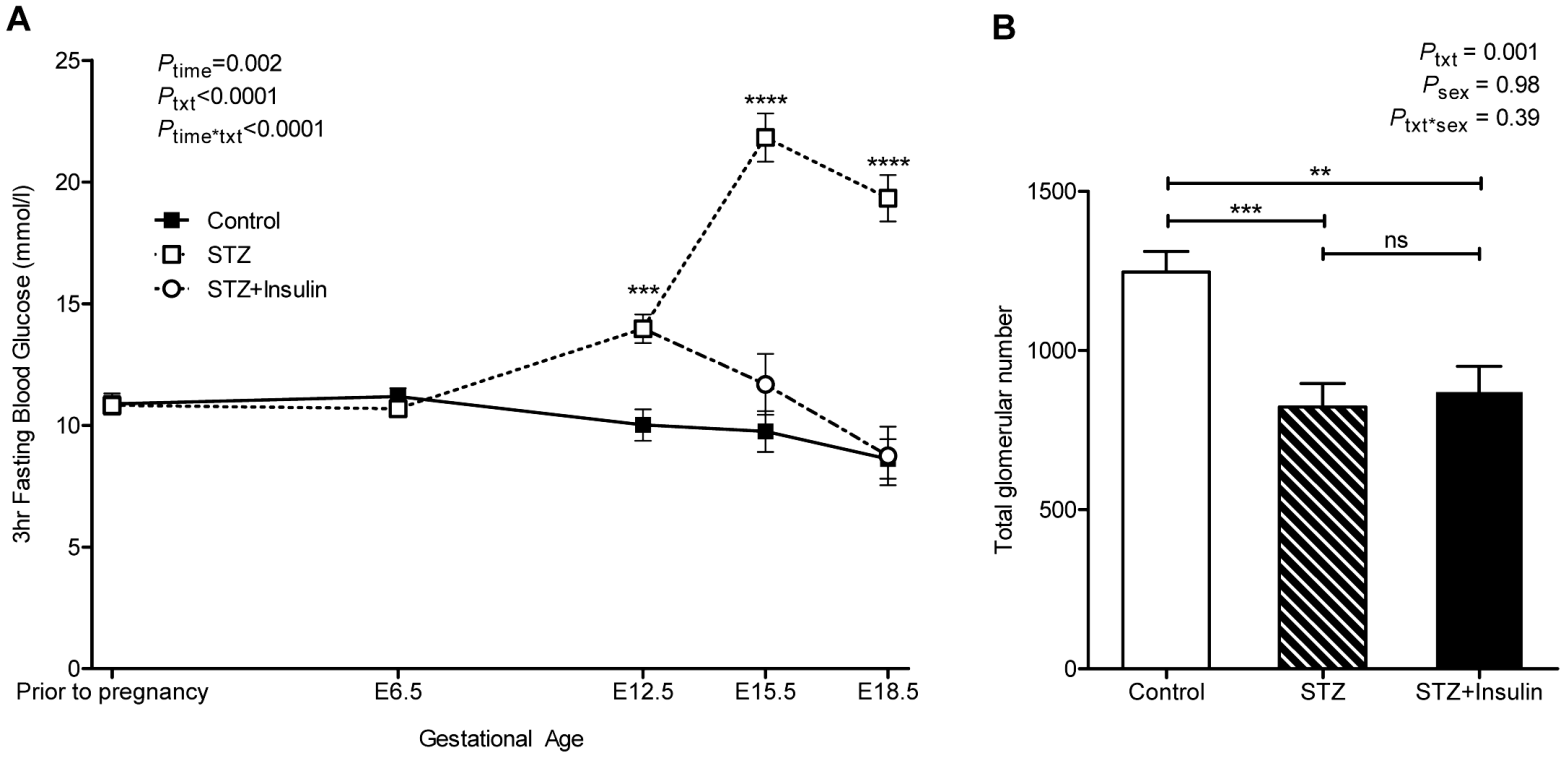
Maternal blood glucose concentrations and glomerular number in offspring of E18.5 cohort. (A) 3 hour fasting blood glucose concentrations of control (solid line, filled square), STZ-treated (dashed line, open square) and STZ+Insulin-treated (dashed line, open circle) dams throughout gestation. (B) Total glomerular number in kidneys of control (clear bar), STZ-treated (striped bar) and STZ+Insulin-treated (solid bar) embryos. Glucose analysis by repeated measures two-way ANOVA for maternal STZ and insulin treatment and time followed by Fishers LSD post hoc analysis; (n)  =  control (11), STZ (8), STZ+Insulin (5). Glomerular number analysis by two-way ANOVA for maternal STZ and insulin treatment and offspring sex followed by Fishers LSD post-hoc analysis; (n)  =  control (7 litters comprising 20 kidneys), STZ (6 litters comprising 14 kidneys), STZ+Insulin (5 litters comprising 10 kidneys). Values are mean±SEM. **p<0.01, ***p<0.001 ****p<0.0001 *vs*. control.

Similar to the E14.5 cohort, maternal blood glucose concentrations did not differ between control (n = 11) and STZ-treated dams (n = 13) prior to pregnancy (*p* = 0.91) or at E6.5 (*p* = 0.29) (**Figure 2A**). Blood glucose concentrations of STZ-treated dams were significantly greater than control values at E12.5 (*p* = 0.0002). Blood glucose concentrations remained significantly higher in STZ-treated dams (n = 8) at E15.5 (*p*<0.0001) and E18.5 (*p*<0.0001). The administration of insulin from E13.5 to STZ-treated dams (n = 5) significantly reduced maternal blood glucose levels to a concentration that was comparable to controls by E15.5 (*p* = 0.21) and at E18.5 (*p* = 0.93) (**Figure2A**). Maternal glucose AUC was significantly different across all treatment groups (control 198.85±2.40 mmol/l.day; STZ 260.48±8.87 mmol/l.day; STZ+Insulin 229.58±2.28 mmol/l.day, *p*<0.0001).

For the E18.5 cohort there was no difference in litter size across the three treatment groups (control 6.7±0.8 embryos; STZ 4.5±0.8 embryos; STZ+insulin 5.4±0.9 embryos, *p* = 0.13). However post-hoc analysis indicated that litters from STZ-treated dams had significantly fewer live embryos than control dams (control 6.6±0.7 embryos; STZ 3.9±0.7 embryos, *p* = 0.02). No difference was detected in the number of resorptions (control 0.5±0.2 resorptions; STZ 1.9±0.6 resorptions; STZ+insulin 1.4±0.5 resorptions, *p* = 0.055) or dead embryos (control 0.1±0.1 embryos; STZ 0.6±0.3 embryos; STZ+insulin 0.4±0.2 embryos, *p* = 0.12) at E18.5.

Post-hoc analysis found embryos of STZ-treated dams were 37% lighter in weight (*p*<0.0001), 20% shorter in crown-rump length (*p*<0.0001) and 14% shorter in head width (*p*<0.0001) than control embryos at E18.5 (**Table 1**). Comparably, STZ+Insulin-treated embryos were smaller than control embryos for weight (38% lighter, *p*<0.0001), crown-rump length (21% shorter, *p*<0.0001) and head width (10% shorter, *p* = 0.004). Body weight, crown-rump length and head width did not differ between STZ-treated and STZ+Insulin-treated embryos. STZ-treated embryos had a 39% lighter placental weight than control embryos (*p*<0.0001). Placental weight of STZ+Insulin-treated embryos was 32% lighter than control embryos (*p* = 0.0007) and did not differ to STZ-treated embryos (*p* = 0.41). Sex was found to have only a significant effect on placental weight, with males having heavier placental weights than females (*p* = 0.04) and there was no treatment*sex interaction.

At E18.5, kidneys of STZ-exposed embryos had 34% fewer nephrons than control kidneys (*p* = 0.0007) (**Figure 2B**). Kidneys of STZ+Insulin-treated embryos had 30% fewer nephrons than control kidneys (*p* = 0.003). Nephron number did not differ between kidneys of STZ and STZ+Insulin-treated embryos (*p* = 0.67).

Spearman's rank coefficient showed that fetal bodyweight, maternal STZ and insulin treatment and maternal glucose AUC had strong associations with glomerular number at E18.5 (**Table 3**). In a stepwise multiple regression model, fetal bodyweight, maternal STZ and insulin treatment and maternal glucose AUC were found to predict glomerular number (R^2^ = 0.72, *p*<0.0001), with fetal bodyweight contributing the most to the model (β = 0.93, *p*<0.0001) above that of maternal STZ and insulin treatment (β = 0.04, *p* = 0.76) and maternal glucose AUC (β = 0.06, *p* = 0.71). When variables were considered independently, fetal body weight was the only statistically significant factor (R^2^ = 0.72, *p*<0.0001).

**Table 3:** Spearman's rank coefficients: associations between glomerular number and independent variables at E18.5.

	Bodyweight	Treatment	Glucose AUC
**Treatment**	−0.74		
	(*p*<0.001)		
**Glucose AUC**	−0.80	0.73	
	(*p*<0.001)	(*p*<0.001)	
**N_glom_**	0.86	−0.71	−0.68
	(*p*<0.001)	(*p*<0.001)	(*p*<0.001)

Spearman's Rho (top value), *p* value (bottom value). Negative values indicate a negative association. AUC  =  area under the curve. N_glom_ =  total estimated glomerular number.

### Malformations of the kidney and urinary tract in offspring of diabetic dams

**Figure 3:**
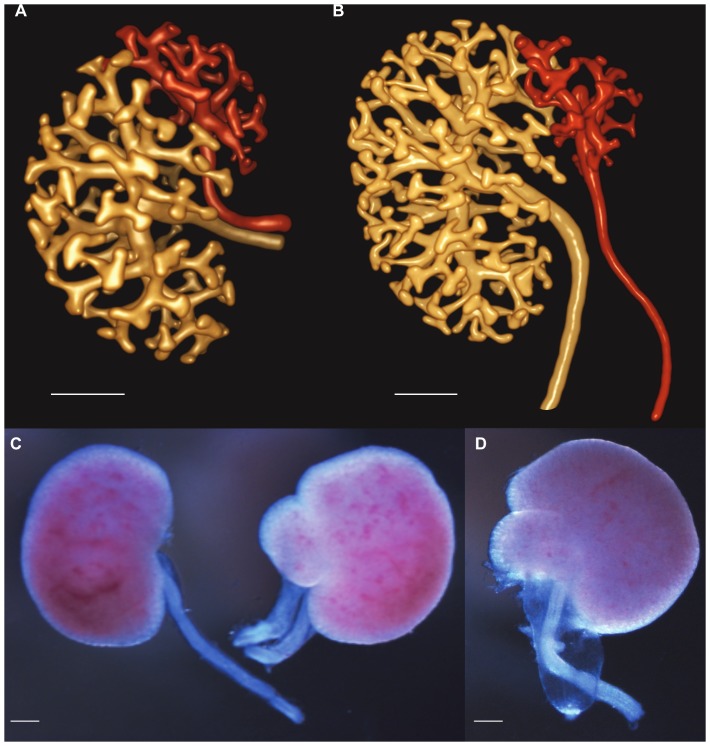
Congenital abnormalities in offspring of mothers with diabetes. (A, B) Rendered OPT images of duplex ureter at E14.5. Note that each ureter is associated with a separate region of the ureteric tree. (C) Duplex ureter at E18.5 compared with control kidney on the left. (D) Hydroureter at E18.5. Scale bar denotes 200 µm.

Interestingly, 26% of offspring of diabetic dams demonstrated renal and urinary tract abnormalities. This was observed as duplex ureter and duplicated collecting duct systems at E14.5 and E18.5 (**Figures 3A–C**), and with hydroureter at E18.5 (**Figure 3D**). Renal malformations equally affected male and female embryos, and were evident in both STZ and STZ+Insulin treated embryos. No urinary tract malformations were observed in control offspring.

## Discussion

This is the first study to apply an *ex vivo* method to analyse development of the ureteric tree of embryos exposed to hyperglycemia, and confirms branching morphogenesis is markedly reduced in hyperglycemia. Our use of OPT and parameterisation of the ureteric tree has shown that kidneys of diabetic pregnancy at E14.5 have approximately half the number of ureteric tips and branch points, a reduced tree length and volume, and fewer branch generations compared with controls. We also report shorter branch segments for the middle branch generations of STZ-treated kidneys, which may correspond to a cumulative disruption in branching morphogenesis as maternal glucose concentrations increase. While no difference in Theiler staging was found between embryos of diabetic and control pregnancies it is possible kidneys of STZ-diabetic embryos are developmentally delayed. Ureteric tree development may resemble that of earlier staged embryos and warrants further investigation.

Our findings regarding ureteric tree development support those of Kanwar et al. [19] and Amri et al. [16] who reported branching dysmorphogenesis and inhibition of ureteric arborisation in metanephroi cultured in the presence of a high concentration of glucose. In contrast, Zhang et al. [20] observed a stimulatory effect of transient high glucose on ureteric branching *in vitro*. While metanephric organ culture has proven a major research tool in developmental nephrology, cultured kidneys are unnaturally flattened, avascular and grow at a relatively slow rate; factors which affect and distort ureteric branching morphogenesis [22], [23]. Inconsistencies between findings from previous studies may be due to differences in media and length of culture time. While it is plausible that short term exposure to high glucose may stimulate branching as observed by Zhang et al. [20], extended periods of exposure, as observed in the present study in diabetic pregnancy, result in a different outcome. As this study and that of Zhang et al. [20] are described in mice on the C57Bl6 background, discrepancies between the two are most likely explained by differences in methodology (*in vivo* STZ-induced diabetes in the dam vs. *in vitro* kidney explants cultured in high glucose) rather than strain differences.

Our findings demonstrate that exposure to hyperglycemia leads to a deficit in nephron endowment at E18.5 as assessed by an unbiased stereological technique. The present study confirms the findings of Amri et al. [16] and Tran et al. [17] who used the acid maceration method and disector/fractionator technique, respectively. Collectively we demonstrate that diabetic pregnancy impairs nephrogenesis in proportion to the magnitude of maternal glycemia. Deficits in nephron endowment in offspring of diabetic pregnancy have been described across a range of species and strains: Sprague Dawley rat (Amri et al. 1999), Wistar rat (Rocha et al. 2005), Swiss mouse (Cunha et al. 2008) and *Hoxb7-GFP* mouse on a C57Bl6 background (Tran et al. 2007). However the findings of Cunha et al. [18] are perhaps questionable as they observed no difference in nephron number between control and STZ-treated mice after the completion of nephrogenesis at PN7 yet reported a 20% reduction at PN21.

We report altered kidney development in growth-restricted offspring of diabetic pregnancy. Human maternal diabetes is typically associated with infant macrosomia and elevated birth weight. However, in a subset of women with severe hyperglycemia, poorly controlled diabetes, vascular complications or excessive insulin administration, intrauterine growth restriction is a frequent observation [24], [25], [26], [27]. In rodent models of diabetic pregnancy, STZ is frequently used and depending on the degree of hyperglycemia may lead to a range of alterations in offspring growth. Both normal and large for gestational age pups [16], [28], [29], [30] have been observed in mildly diabetic dams, however growth restriction is a common outcome in severely hyperglycemic STZ-treated dams [17], [31]. In the latter instance, this is due to profound fetal hyperglycemia and hyperinsulinemia associated with fetal pancreatic exhaustion. In the present study STZ-treated dams exhibited severe fasting hyperglycemia and their offspring were hyperglycemic (measured in amniotic fluid at E14.5) and growth restricted. Kidney development in large for gestational age offspring of diabetic pregnancy has yet to be investigated. This may relate to the difficulty in obtaining macrosomic pups in rodents where fat composition at birth is low (2% fat) compared to humans (∼15% fat). However, considering the strong linear relationship we observed between fetal growth and nephron endowment the effect of maternal diabetes in macrosomic offspring would be of particular interest. Despite restoring glucose levels to within the range of control dams from E15.5, insulin treatment did not normalise fetal growth of STZ+insulin treated dams. This may be due to the timing of insulin treatment after hyperglycemia onset considering insulin mini-pumps were implanted at E13.5, seven days after STZ administration. Studies utilising immediate insulin treatment following STZ administration, in contrast, report normal embryo growth [32], [33]. It is therefore likely that the persistent deficit in nephron number at E18.5 observed in STZ+insulin exposed embryos is related to the late instigation of glycemic control. Embryos exposed to diabetes had a marked deficit in ureteric branching morphogenesis at E14.5. As nephron induction occurs at the site of nascent ureteric tips it is likely that a low nephron number was already established prior to insulin treatment at E13.5. Insulin was administered at this time point to determine if the effect on the developing kidney is principally due to altered branching morphogenesis (which largely occurred prior to instigation of insulin treatment) or nephron induction/maintenance (which primarily occurred post commencement of insulin treatment). While we did not expect complete normalisation of nephron endowment at E18.5 in this group, if the process of nephrogenesis was indeed retarded by hyperglycemia then we might predict that normalizing glycaemia would result in some increase in the nephron endowment. As this was not observed, we conclude that the process of branching morphogenesis was severely affected giving rise to a deficit in ureteric tip number and thus sites for nephron formation. An alternative explanation is that insulin therapy was administered too late after diabetes induction and late glycemic control, initiated past the time-point when ureteric branching morphogenesis is at its peak, could not restore these deficits. The hypothesis that impaired ureteric branching is central to the nephron number deficit is supported by Chen at el. [34] who found insulin treatment to partially normalise nephron number in neonates of STZ-treated dams where diabetes was induced later in gestation at E13 followed by insulin implantation at E15. This protocol partially restored maternal blood glucose concentrations from a 5-fold to 2.5-fold elevation compared with controls, normalised neonatal bodyweight to within control values and reduced the nephron deficit by half [34], [35].

Infants of gestational diabetic and overt diabetic pregnancies have an elevated risk of anomalies of the urinary tract [14], [36], [37]. We observed malformations of the urinary tract in embryos of diabetic pregnancy, further suggesting that early ureteric branching morphogenesis and possibly ureteric budding are primary processes targeted in hyperglycemia. These abnormalities included duplex ureters and hydroureter. Together the findings highlight that the longer the window of exposure to diabetes the more adverse the consequences are to fetal and renal development, yet may also indicate that hyperglycemia exposure during specific, shorter windows of development (i.e. when branching of the ureteric tree is maximal) may also be detrimental to kidney development.

The effects of STZ treatment in this study are ascribed to changes in maternal pancreatic function, but direct effects of STZ on the fetus should also be considered. Previously, the addition of STZ (1 mg/ml) to cultured rat embryos was found to reduce their viability and growth (Deucher et al. 1977). This dose was administered to reflect the dose given to an adult rat to induce diabetes and likely does not reflect the level of STZ the fetus is exposed to *in vivo*. In the rhesus monkey, STZ has been reported to cross the placenta with relatively low concentrations reaching the fetus (Reynolds et al. 1974), but data for rodents is lacking. While we cannot rule out the secondary effects of STZ on fetal growth, given the short half-life of STZ (∼15 min) and the small volumes reaching the fetus we believe it is unlikely that STZ exerts a toxic effect on the fetus. This can be further extended to the developing kidney. Tran et al (2008) examined kidney size and the number of glomeruli in fetuses of STZ-exposed dams that went on to develop or not develop diabetes. They found renal damage to be independent of STZ administration or length of STZ exposure but attributable to the level of maternal hyperglycemia. This finding is supported by Amri et al. (1999) who reported a comparable nephron deficit in offspring of a glucose infusion model in the pregnant rat and in offspring of STZ-induced diabetes. STZ has a serum half-life of 15 minutes [38] and following STZ treatment at E6.5, E7.5 and E8.5 in the present protocol it is expected to be cleared from the maternal circulation before fetal metanephric development commences at E10.5.

In our model of growth restriction in diabetic pregnancy it is difficult to discriminate the effect between low birth weight and high glucose as the driving force of altered kidney development. Regression analysis identified fetal growth as the strongest predictor of kidney development in diabetic pregnancy, and is consistent with published literature. Human studies have identified a strong linear relationship between birth weight and nephron number [39], [40], [41]. Animal models of spontaneous [42], [43] and induced growth restriction [42], [44], [45] further support this association. In rodents, high glucose exposure is reported to alter kidney morphology and lead to a nephron deficit in growth restricted and normal birth weight offspring [16], [17]. The direct effect of high glucose on the kidney has been linked to aberrant Pax-2, NF-κβ and p53 signaling pathways, nascent nephron apoptosis [17], [34] and the altered expression of extracellular matrix glycoproteins [46]; processes mediated by elevated reactive oxygen species (ROS) generation. While infants of diabetic pregnancy have an increased risk of renal and urinary tract malformations [15], [36], [37], less severe outcomes such as altered nephron endowment in persons exposed to intrauterine hyperglycemia have not been described.

Pregnant women are typically not screened for diabetes until 28-30 weeks gestation. As kidney development begins at 5 weeks gestation with the full complement of nephrons reached by 36 weeks, the developing fetal kidney may be unknowingly exposed to a lengthy window of hyperglycemia. Impaired kidney development may therefore already be established and pharmacological interventions such as insulin therapy could have limited potential to mediate fetal kidney development, if the outcomes of this study are transferrable to humans. Impaired kidney development (by altered ureteric branching morphogenesis, nephrogenesis, or both) may lead to a permanent nephron deficit and a predisposition to hypertension and chronic renal disease, a hypothesis first enunciated by Brenner and colleagues [47]. While offspring were not followed into adulthood, we can predict that offspring may display a range of long-term metabolic defects as reported in a small number of animal [28], [35], [48] and human studies [49], [50], [51].

In conclusion, we report ureteric branching morphogenesis and nephrogenesis to be adversely affected in offspring exposed to STZ-induced diabetic pregnancy. We present the first study to examine *ex vivo* and in 3D the ureteric tree of embryos of diabetic pregnancy, and report a marked deficit in ureteric branching morphogenesis. We hypothesize that this early alteration in ureteric tree architecture gives rise to the nephron deficit observed in late gestation. Glycemic control which normalized maternal glucose levels to that of control values did not prevent embryo growth restriction or a nephron deficit prior to birth, and highlights the detrimental effect of hyperglycemia in pregnancy on kidney development. This suggests that late insulin therapy would not be useful in preventing aberrant kidney growth. This is particularly important for women identified with diabetes relatively late in gestation as kidney development is well underway, and potentially irreversible deficits in branching morphogenesis and nephrogenesis may be established. This adds impetus to the importance of vigilant glucose monitoring throughout pregnancy as it may set the scene for poor kidney health and long-term consequences in the offspring.

## Materials and Methods

### Animals

Experimentation was performed on 8-12 week old C57BL/6J mice (Monash Animal Services, Victoria, Australia). All animal handling and experimental protocols were approved by the Animal Ethics Committee of Monash University (SOBS A/2010/07) and conformed to the guidelines of the National Health and Medical Research Council of Australia. Mice had *ad libitum* access to standard chow and water with a 12 hour light/dark cycle. Mice were mated overnight. The presence of a vaginal plug the following morning indicated embryonic day 0.5 (E0.5).

### Diabetes Induction

Maternal diabetes was induced by the intraperitoneal administration of streptozotocin (STZ) (Sigma-Aldrich, Castle Hill, Australia) in 0.1 M sodium citrate buffer. STZ was administered for 3 consecutive days commencing at E6.5 at doses of 100, 100 and 80 µg/g bodyweight. Control mice received intraperitoneal injections of 0.1 M sodium citrate buffer for 3 days from E6.5. Maternal blood glucose concentration (mmol/l) was measured prior to mating and throughout pregnancy by cheek bleed following a 3 hour fast (Accu-Chek Go Blood Glucose Monitor, Roche Products, Dee Why, NSW, Australia). The glucose concentration of amniotic fluid at E14.5 was measured on the same glucometer. Maternal glucose concentrations across gestation were estimated by plotting glucose concentrations (mmol/l) against time (days) and quantifying the area under the curve (AUC, mmol/l.day).

### Insulin Treatment

A proportion of hyperglycemic dams received insulin to form a glycemic control group (n = 5). Dams at E13.5 were surgically implanted under isoflurane anesthesia with an osmotic mini-pump (model 1007D, Alzet, Durect Co., Cupertino, CA, USA) filled with NovoRapid insulin aspart (100 U/ml, Novo Nordisk, Baulkham Hill, NSW, Australia) at a 1:10 dilution in saline with an infusion dose of 0.5 µl/hr. Control animals underwent sham surgery that followed the protocol as outlined above bar the implantation of the mini-pump (n = 4). Tissue was collected at E18.5 for animals in the glycemic control group.

### Tissue Collection

Pregnant mice were dissected at E14.5 and E18.5 to form two separate cohorts. Embryo development was verified by Theiler staging criteria [52]. Embryos and placentas were weighed and measures of crown-rump length and head diameter recorded with digital micro-calipers. Embryos were sexed (based on gonad appearance) and metanephroi removed and fixed in 4% paraformaldehyde (PFA) in phosphate buffered saline (PBS) (Sigma-Aldrich, Castle Hill, NSW, Australia).

### Analysis of Ureteric Tree Development: Optical Projection Tomography

The ureteric epithelium of E14.5 kidneys was wholemount fluorescently immunostained, optically cleared and imaged by OPT as based on the protocol of Short and Smyth [53]. Metanephroi were transferred to methanol and subject to 3 freeze-thaw cycles to assist antigen retrieval [54]. Kidneys were rehydrated, blocked, and incubated in E-cadherin primary antibody (1:100 dilution, rat E-cadherin monoclonal antibody; Invitrogen, Mulgrave, VIC, Australia). Kidneys were washed and then incubated in secondary antibody (1:400 dilution, Alexa Fluor 555 goat anti-rat IgG; Invitrogen, Mulgrave, VIC, Australia). Stained E14.5 kidneys were embedded in 1% low melting point agarose, dehydrated in methanol and cleared in benzyl alcohol/benzyl benzoate (1:2 mixture) (Sigma-Aldrich, Castle Hill, NSW, Australia). Immunostained kidneys were imaged in a Bioptonics 3001 OPT scanner and OPT tomographic data were reconstructed using N-Recon software (SkyScan, Kontich, Belgium). Drishti software (v2.0, Australian National University, ANUSF VizLab, Canberra, ACT, Australia) was used to visualise and render reconstructed 3D data sets (see provided Supplementary Information Video S1).

Quantitative assessment of the ureteric tree was performed using Tree Surveyor software (version 1.0.8.20 [55]) from which branch point number, ureteric tip number, tree length and tree volume were automatically obtained. Kidneys with duplex ureters and collecting duct systems were excluded from analysis.

### Estimation of Glomerular Number: Histochemistry and Stereology

Total glomerular number was determined using an unbiased stereological method as previously described [21]. Briefly, metanephroi were embedded in paraffin and exhaustively sectioned at 5 µm. 10 evenly spaced section pairs were systematically sampled and histochemically stained with the lectin peanut agglutinin (PNA) to localise the plasma membrane of glomerular podocytes. Sections were counterstained with hematoxylin. Section pairs were used to estimate PNA-positive developing nephrons using the physical disector/fractionator combination [21]. Kidneys with duplex ureter and hydroureter were excluded from analysis.

### Statistical Analysis

Data were analysed using SPSS (version 19, SPSS Inc., USA) and GraphPad Prism (version 5, GraphPad Software, Inc., USA). Glucose levels were analysed by two-way repeated measures ANOVA with maternal STZ and insulin treatment and time as main effects, followed by Fishers LSD *post hoc* analysis. Litter size was analysed by independent samples *t-*test at E14.5 and one-way ANOVA at E18.5. Measures of embryo growth and kidney development were analysed by a two-way repeated measures ANOVA for maternal STZ and insulin treatment and offspring sex as main effects, and incorporating a mixed linear model to account for any intra-litter bias [56]. Spearman's rank co-efficient with Bonferroni correction was used to measure associations between kidney development and independent variables of interest (fetal weight, maternal STZ and insulin treatment, maternal glucose AUC and offspring sex). Spearman's rank coefficient and multiple regression analysis were performed using STATA (version 8.0, Stata Corporation, USA). Throughout the paper *n* refers to the number of dams or litters and not the total number of pups. No difference in growth or kidney development was found between control and control-sham embryos at E18.5 therefore the two groups have been pooled to conserve power. Offspring sex was incorporated as a variable in all analyses and had no statistically significant effect unless otherwise stated. Data are presented as mean ± standard error of the mean (SEM). *p*<0.05 was considered statistically significant.

## Supporting Information

Video S1 **Reconstructed 3D data set of E15 mouse kidney.**
(M4V)
